# Production of Fish Protein Hydrolysates from *Scyliorhinus canicula* Discards with Antihypertensive and Antioxidant Activities by Enzymatic Hydrolysis and Mathematical Optimization Using Response Surface Methodology

**DOI:** 10.3390/md15100306

**Published:** 2017-10-10

**Authors:** José A. Vázquez, Maria Blanco, Agueda E. Massa, Isabel Rodríguez Amado, Ricardo I. Pérez-Martín

**Affiliations:** 1 Grupo de Reciclado y Valorización de Materiales Residuales (REVAL), Instituto de Investigacións Mariñas (IIM-CSIC), r/Eduardo Cabello, 6, Vigo-36208 Galicia, Spain; 2 Grupo de Bioquímica de Alimentos, Instituto de Investigacións Mariñas (IIM-CSIC), r/Eduardo Cabello, 6, Vigo-36208 Galicia, Spain; mblanco@iim.csic.es (M.B.); ricardo@iim.csic.es (R.I.P.-M.); 3 Instituto Nacional de Investigación y Desarrollo Pesquero (INIDEP), Paseo Victoria Ocampo N°1 Escollera Norte, C.C.175, 7600 Mar del Plata, Argentina; aguedamassa@inidep.edu.ar; 4 Consejo Nacional de Investigaciones Científicas y Técnicas (CONICET), Rivadavia 1917, 1033 Buenos Aires, Argentina; 5 Departamento de Química Analítica y Alimentaria, Facultade de Ciencias de Ourense, Universidade de Vigo, Campus As Lagoas s/n, 32004 Ourense, Spain; sabelara@uvigo.es

**Keywords:** Common Fishery Policy, fish discards, *Scyliorhinus canicula* muscle by-products, fish protein hydrolysates, enzyme hydrolysis, response surface methodology, antihypertensive activity, antioxidant activity

## Abstract

Fish discards are of major concern in new EU policies. Alternatives for the management of the new biomass that has to be landed is compulsory. The production of bioactive compounds from fish protein hydrolysates (FPH) has been explored in recent years. However, the viability of *Scyliorhinus canicula* discards, which might account for up to 90–100% of captures in mixed trawler, gillnet, and longline industrial fisheries, to produce FPH from the muscle with bioactivities has still not been studied in terms of the optimization of the experimental conditions to enhance its production. The effect of *pH* and temperature on the hydrolysis of the *S. canicula* muscle was mediated by three commercial proteases using response surface methodology. Temperatures of 64.6 °C and 60.8 °C and pHs of 9.40 and 8.90 were established as the best hydrolysis conditions for Alcalase and Esperase, respectively. Optimization of the best conditions for the maximization of antihypertensive and antioxidant activities was performed. Higher Angiotensin-converting enzyme (ACE) activity was found with Esperase. The *pH* optimum and temperature optimum for antioxidants were 55 °C/pH8.0 for ABTS/DPPH-Esperase, 63.1 °C/pH9.0 for DPPH-Alcalase, and 55 °C/pH9.0 for ABTS-Alcalase. No hydrolysis was detected when using Protamex.

## 1. Introduction

The new European marine policy framework that aims to achieve a sustainable use of marine resources to ensure high long-term fishing yields has been included in the last reform of the Common Fishery Policy, along with new guidelines to reduce the wasteful practice of discarding by the introduction of a landing obligation (Regulation EU N° 1380/2013). The need of implementing alternatives for the management of the new biomass that should be landed is therefore compulsory. Such an approach is also consistent with the Blue Growth initiative of European Horizon 2020, which aims to ensure and support the sustainable growth of natural marine resources. The success of this blue strategy depends on the development of a blue technology focused on the exploration of bioactive compounds obtained from marine organisms including discards and by-products with potential interest in the food/feed/pharmaceutical/cosmetical industries.

*Scyliorhinus canicula* (small-spotted catshark) is generally captured as a non-target (by-catch) alongside the target species in mixed trawler, gillnet, and longline industrial fisheries, with very high discard rates reaching 90–100% [1]. Discards of this species in the Cantabrian Sea and North Portugal waters account for approximately 1200 tn/year [1,2]. The huge amount of potential discards of this species, together with the elevated percentage of muscle fraction in *S. canicula* (43.56 ± 3.78%), makes the hydrolysis of muscle proteins an excellent alternative to efficiently upgrading this biomass [2]. The preparation and characterization of fish protein hydrolysates (FPH) covering different species, enzymes, or hydrolysis conditions have been extensively studied [3]. The presence of essential nutrients and bioactive compounds in FPH transforms them into high value added products with potential to be used in a wide range of industrial sectors such as food [4,5] or pharmacy [6]. Lipid oxidation and cardiovascular diseases are of particular concern for the food industry and international public health organizations, respectively. FPH from other fish substrates and by-products have been cited to possess antihypertensive and antioxidant bioactivities [7,8,9,10]. Only one reference about the production of enzymatic hydrolysates with biological properties from *S. canicula* has been reported [11]. However, the optimization of the experimental conditions to enhance the production of FPH and bioactivities from small-spotted catshark muscle has not been still explored.

In the present work, the effect of *pH* and temperature on the hydrolysis of *S. canicula* muscle mediated by three commercial proteases, Alcalase, Esperase, and Protamex, was studied using response surface methodology (RSM). Optimization of the best conditions for the maximization of hydrolysis degree and the production of bioactive peptides with antihypertensive and antioxidant properties was performed. These objectives are consistent with the current policy framework above mentioned.

## 2. Results and Discussion

In previous reports, different parts of *S. canicula* by-products have been valorised obtaining various products and biocompounds of medium-high added value. Cartilages from fins, skeletons, and heads were processed to isolate chondroitin sulphate [12]; marine peptones used as microbial nutrients were recovered from viscera [13], proteolytic activities including trypsin analogues were extracted from pancreas [14], and collagen and derivatives were produced from skins [15]. The corresponding muscle from *S. canicula* discards that cannot be used for human consumption may be also a valuable substrate for the production of FPH and bioactive peptides.

### 2.1. Composition of S. canicula Muscle

The average (±SD) chemical composition of muscle from *S. canicula*, expressed as a percentage of wet weight, was 77.79 ± 0.70, 1.11 ± 0.06, 22.19 ± 0.46, and 0.35 ± 0.06 of moisture, ash, protein, and lipids respectively. The protein content was higher, the lipid content lower, and the ash content similar to values reported for the edible portion of most common finfish and crustacean species [16]. When compared to other elasmobranchs, protein and ash content of small-spotted catshark were higher (except when compared to smooth hound which presented a higher content of ashes), while lipid content was lower [17,18]. The low lipid content found in the flesh of small-spotted catshark muscle has a positive impact on the overall quality of this species (low rancidity, low oxidation, and enhanced texture of the flesh).

### 2.2. Hydrolysis Process and Production of FPH

Three commercial proteases, Alcalase, Esperase, and Protamex, were selected for the hydrolysis of the present by-products. They were chosen due to their excellent ability to digest several substrates from different fish by-products and their low cost when applied in industrial processes [3,19,20]. The experimental and predicted profiles of hydrolysis degrees, executed under the conditions specified in Table S1, are displayed in Figures S1 and S2. In various combinations of *pH* and temperature, no detectable degrees of hydrolysis were observed (for example, highest *pH* in Alcalase or lowest temperature in Esperase). Additionally, the results for Protamex catalysis were not presented since no hydrolysis was detected and therefore discarded for the evaluation of bioactivities. This last issue was unexpected because Protamex has reported good hydrolytic ability on muscle of, among others, catfish, small croaker, and Pacific hake [21,22]. It may be due to the possible presence of inhibitor compounds of Protamex activity in the muscle discards of *S. canicula*.

Experimental data of enzyme hydrolysis were accurately modelled by Weibull equation [5] with coefficients of determination ranging from 0.951 to 0.999 (Table 1).

The experimental and theoretical profiles were almost indistinguishable. In almost all cases the maximum hydrolysis was reached at 2–4 h (stationary phase); therefore, *S. canicula* kinetics could be shorted and the ratio S:L and enzyme concentration could be also reduced. In fact, using a S:L ratio of (1:2), Alcalase concentration of 0.5% (*v*/*w*) and run kinetics for 4 h at 60 °C/*pH* = 8.60, hydrolysis of 24%, was achieved (data not shown). The robustness of Weibull equation to predict the present patterns was also confirmed in all kinetics (*p* < 0.001 from Fisher *F*-test) and the numerical parameters were always statistically significant (Student *t*-test). The validity of the Weibull equation proposed has been again revealed. Habitually used for the modelling of dose-response curves, animal growths, and chemical and enzymatic reactions without mechanistical basis [7,23], that mathematical model has been recently applied to the description of enzyme proteolysis of fish cartilages [24] and squid pen [25] with excellent outcomes. Two parameters (*H_m_* and *v_m_*) from equation [5] were selected as responses (dependent variables) to assess the effect of *pH* and *T* on proteolysis.

**Figure 1:**
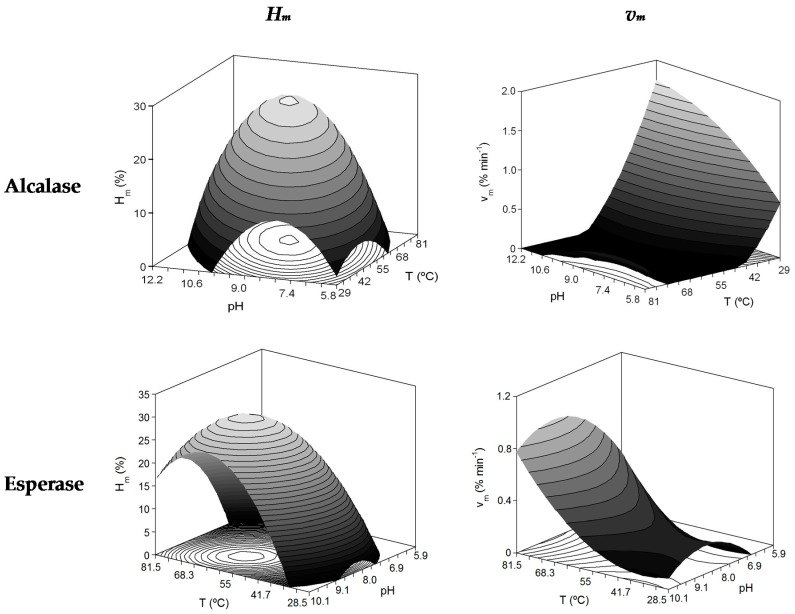
Theoretical response surfaces describing the combined effects of the temperature and *pH* on the Weibull parameters obtained by Alcalase and Esperase proteolysis of *S. canicula* muscle and summarized in Table 1.

Figure 1 illustrates the plots corresponding with the 3D-surfaces predicted by the second-order equations as a function of *T* and *pH* (Equation (1)) using RSM and the multivariable analysis summarized in Table 2.

The values for the non-hydrolysis detected (NHD) were assumed as zero. The coefficients of determination of the theoretical surfaces ranged from 0.413 to 0.943. Very poor proportion of variability was explained for the case of *v_m_* with Esperase (41%), but remarkable fittings were obtained in the other responses (>69%). The consistence of the polynomial equations was also demonstrated in all situations (*F1* and *F2* were always confirmed).

The coefficients present in the equations defined in Table 2 indicated that the quadratic effects of both variables were statistically significant in all cases and always with negative sign for *pH*. The quadratic term of *T* was also negative for the maximum degree of hydrolysis (*H_m_*) and the interaction *pH* × *T* was significant for this parameter. Thus, the theoretical surfaces show the typical form of convex dome with clear maximum at unique values of *pH* and *T*. In order to calculate those optimal levels, mathematical optimization using numerical derivation of empirical equations was performed. The optima values of both variables (*pH_opt_* and *T_opt_*) and the corresponding maximum value for each response (*Y_max_*) are also represented in Table 2. Temperatures of 64.6 °C and 60.8 °C and pHs of 9.40 and 8.90 were established as the best hydrolysis conditions for Alcalase and Esperase, respectively. These levels are much higher than commonly applied (50 °C/*pH* = 7.0–8.0) in the production of FPH production using other fish substrates but without previous optimisation of both variables [8,25]. Higher capacity of hydrolysis of Esperase (30.7%) in comparison to Alcalase (26.3%) was predicted under the optimal conditions mentioned (Table 2). Our *H_m_* data for Alcalase are in general similar or higher to other species as Yellow stripe trevally, Pacific whiting, Cape hake and horse mackerel [8,10,26]. However, greater degree of hydrolysis (44%) was described for salmon-FPH using an acid fungal protease [27] and anchovy sprat-FPH formulated with Papain or Bromelain [25]. The results of Esperase can not be compared with literature data because our study is the first report addressing the production of FPH catalysed by such endoprotease.

The case of *v_m_* was more heterogeneous; equations described different surfaces depending on the enzyme used, and the highest maximum rate of hydrolysis were achieved at high *pH* and low *T* for Alcalase and high *T* and *pH* = 8.65 for Esperase. Nevertheless, the reliability of the equations is much lower than in the case of *H_m_*. This kinetic parameter (*v_m_*) is not applied in the studies concerning FPH since the modelling of FPH kinetics is habitually inexistent.

### 2.3. Determination of the In Vitro Antihypertensive Activities of the Produced FPH

The samples of hydrolysates at the end of the enzyme process (6 h) were analysed quantifying the ACE activity as percentage of inhibition and as the value of *IC*_50_ [28]. The experimental and expected data from the factorial design are compiled in Tables S2 and S3. The corresponding polynomial equations calculated from such data demonstrated the significant effect of *T* and the non effect of *pH* in three of the four cases evaluated (Table 2). The explanatory capacity of those models was higher for the case of Alcalase (>74%) than Esperase (>50%).

**Figure 2:**
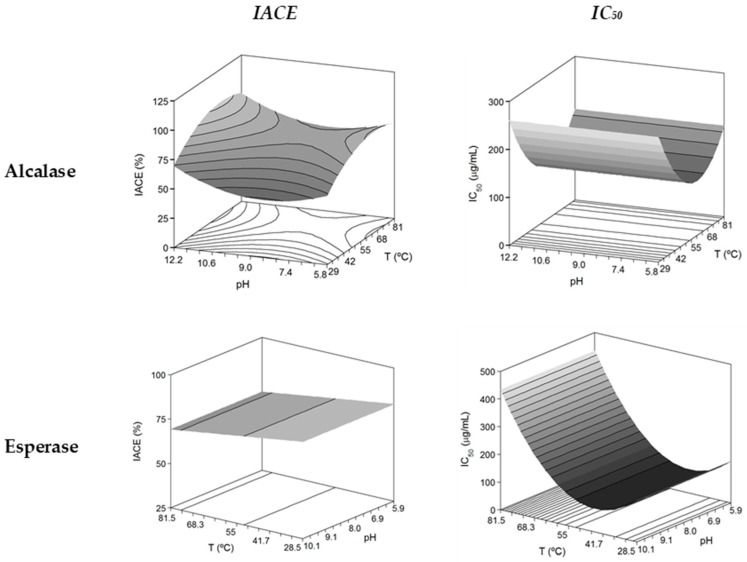
Theoretical response surfaces describing the combined effects of the temperature and *pH* of enzymatic hydrolysis on antihypertensive activities of *S. canicula* muscle hydrolysates and summarized in Table S2.

The multivariate analysis for the experimental data of Esperase predicted a surface in the form of a sheet; only linear coefficient of *T* was significant, producing greater *IACE* values at high temperatures (Figure 2). The case of Alcalase was somewhat more complex with better conditions of operation at high and low temperatures and pHs in a characteristic surface with structure of saddle. The optimal values were 66 °C and *pH* = 11.5.

The theoretical surfaces show similar form in the *IC*_50_-response for both enzymes, that is, constant values for any level of *pH* and concave sheet with a minimum at 59.4 °C for Alcalase and 44.6 °C for Esperase (Figure 2 and Table 2). Thus, lower value of *IC*_50_ (higher activity) was found employing Esperase (83.6 μg/mL) than using Alcalase as catalyst (114.5 μg/mL). The values of *H_m_* in the optimal conditions for maximizing *IC*_50_ can be calculated from the polynomial equations listed in Table 2 and were 25.9% and 23.1% for Alcalase and Esperase, respectively. As it was reported by [29], the presence of shorter peptides (obtained at high degrees of hydrolysis) in blue whiting hydrolysates lead to highest ACE-inhibitory activity (lower values of *IC*_50_); thus, our results are in concordance with that assumption: high *H_m_* values, but not the highest, generated almost the best bioactivities (lower *IC*_50_ values and levels of *IACE* greater than 75%).

In addition, *IC*_50_ data obtained in the current study are much lower (higher antihypertensive capacity) than exhibited by other authors. Values ranging 0.25–7.4 mg/mL were observed for hydrolysates prepared from horse mackerel, sardinelle, blue whiting, trevally and goby by-products, and discards employing various types of proteases [11,17,29,30,31]. Our crude hydrolysates are also more active (83.6 and 114.5 μg/mL) than crude FPH produced from *S. canicula* muscle (302 μg/mL) by the simultaneous combination of subtilisin and trypsin [11]. These authors improved the bioactivite of the hydrolysates through the isolation of peptide fractions of lower molecular weights by size-exclusion chromatography (SEC). Two fractions of 470–1210 Da and <470 Da yielded values of 72 and 27 μg/mL, respectively, and were associated to the presence, among others, of the following peptides: ELVGV, LVAPAN, and VAMPF. These peptides may be responsible for the excellent antihypertensive activities reported here.

### 2.4. Determination of Antioxidant Activities of the Produced FPH

Because the mechanism of inhibition of oxidation process in biological samples is dependent on the chemical features of the antioxidant and the matrix, different types of antioxidant activities were measured in each sample obtained from the experimental design. In the present work, four protocols were applied (DPPH, ABTS, crocin, and *β*-carotene bleaching assays). Experiment data and numerical expected values obtained from second-order equations are summarized in Tables S2 and S3.

**Figure 3:**
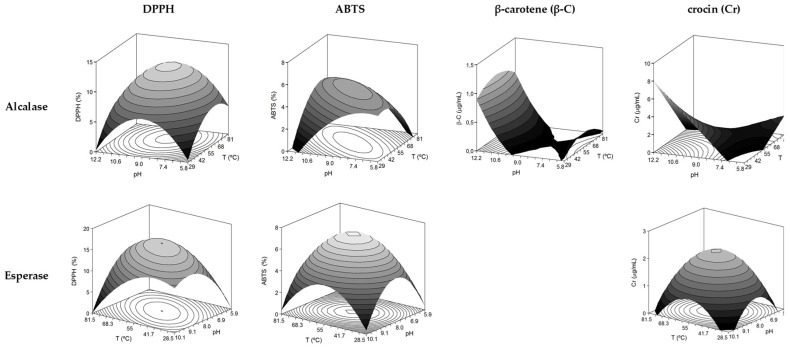
Theoretical response surfaces describing the combined effects of the temperature and *pH* of enzymatic hydrolysis on antioxidant activities of *S. canicula* muscle hydrolysates and summarized in Table S3.

The outcomes of DPPH and ABTS for both enzymes were quite similar: convex domes were the 3D-surfaces expected (Figure 3) due to the negative terms obtained for the quadratic effect of *T* and *pH* (Table 3).

The coefficients of determination were also greater for DPPH than ABTS response. The values of *pH_opt_* and *T_opt_* were found closer to the center of the experimental domain, 55 °C/pH8.0 for ABTS/DPPH-Esperase, 63.1 °C/pH9.0 for DPPH-Alcalase, and 55 °C/pH9.0 for ABTS-Alcalase. DPPH and ABTS maximum values varied among 12.4% and 5.1% for Alcalase hydrolysates and 16% and 7.3% for Esperase hydrolysates. These antioxidant activities are lower than those addressed for FPH formulated with different materials and using several enzymes: horse mackerel and subtilisin [10], red scorpionfish and Flavourzyme [32], or threadfin bream and Papain [33].

The crocin response with Esperase followed an identical pattern in terms of the significant response surface defined by the significant second-order model and the corresponding optima conditions calculated. On the contrary, crocin results for Alcalase displayed the best operatory values when protease was exposed to lower temperature and higher level of *pH*. The hydrolysates generated by Esperase did not show any type of capacity to slow down the *β*-carotene bleaching reaction. Finally, the maximum response for *β*-C and Alcalase was established at 55 °C and high value of *pH*.

## 3. Materials and Methods

### 3.1. S. canicula Discards

Discards from *S. canicula* were obtained in a local market (Vigo, Spain) and stored at −4 °C until use. Specimens were thawed and the muscle tissue removed, mixed thoroughly, ground, separated into different batches, and stored in sealed plastic bags at −20 °C. 

#### Proximate Composition of *S. canicula* Muscle

Muscle was analysed for crude protein (N × 6.25) by Kjeldahl method in a DigiPREP 500 fully automatic steam distillation (SCP Science, Baie-D’Urfe, QC, Canada) and a TitroLine Easy Unit (Metrohm AG, Ionenstrasse, Switzerland). Lipid content was determined by the methodology of [34]. Moisture was determined after heating the sample at 105 °C for 24 h, and ash content was determined after heating the sample 24 h at 550 °C.

### 3.2. Experimental Design

The simultaneous effect of two factors, temperature (*T*) and *pH*, on the enzymatic hydrolysis of *S. canicula* muscle by-products was evaluated by a rotatable second order design [35]. The values of the variables tested for each protease and the procedure of codification-decodification of the variables is summarized in Table S1. The commercial proteases were Alcalase 2.4 L (2.4 Anson Unit/g, AU/g), Esperase 8 L (8 KNovo Protease Unit/g, KNPU/g) and Protamex (Novozymes, Nordisk, Bagsvaerd, Denmark), and the concentration employed was 1% (*v*/*w* of muscle) in all cases. The rest of the experimental conditions were maintained constant: ratio solid:liquid (1:5) and 200 rpm of agitation. The kinetics of hydrolysis were performed in a controlled pH-Stat system with a 100 mL glass-reactor and extended up to 6 h. At the end of hydrolysis, the samples were heated at 90 °C for 15 min to inactivate the proteases and were stored at −20 °C until analysis.

Orthogonal least-squares calculation on factorial design data were used to obtain the empirical equations describing the different dependent variables (hydrolysis kinetic parameters and bioactivities) assessed (*Y*) in function of the independent variables (*T* and *pH*):

(1) $$Y=b_{0}+b_{1}T+b_{2}pH+b_{12}TpH+b_{11}T^{2}+b_{22}pH^{2},$$

where *Y* represents the parameters to be modelled; *b*_0_ is the constant coefficient, *b*_1_ and *b*_2_ are the coefficient of linear effects, *b*_12_ is the coefficient of interaction effect among *pH* and *T*, and *b*_11_ and *b*_22_ are the coefficients of quadratic effects. The Student *t*-test (α = 0.05) was employed to determine the statistical significance of the coefficients. The goodness-of-fit was established as the determination coefficient (R^2^) and the model consistency by the Fisher *F*-test (α = 0.05) using the following mean squares ratios:

the model is acceptable when*F1* = Model/Total error
$F1\geq F_{den}^{num}$
*F2* = (Model + Lack of fitting)/Model
$F2\leq F_{den}^{num}$
*F3* = Total error/Experimental error
$F3\leq F_{den}^{num}$
*F4* = Lack of fitting/Experimental error
$F4\leq F_{den}^{num}$


$F_{den}^{num}$ are the theoretical values to α = 0.05 with the corresponding degrees of freedom for numerator (num) and denominator (den). The equation is acceptable when *F1* and *F2* are validated. *F3* and *F4* were additionally calculated to improve the degree of robustness and consistency of the empirical equations obtained. A Microsoft Excel spreadsheet was employed for the procedures of numerical fittings, coefficient estimates, and statistical evaluations.

### 3.3. Enzyme Proteolysis of Muscle Discards

The hydrolysis degree (*H*, in %) was determined by the pH-Stat method defined by Adler-Nissen [36] by applying the following equation:

(2) $$H=\frac{BN_{b}}{\alpha M_{p}h_{\mathrm{tot}}},$$

where *B* is the volume (mL) of 2 M NaOH consumed during hydrolysis; *N*_b_ is the normality of NaOH; *M*_p_ is the mass (g) of initial protein (N × 6.25); *h*_tot_ is the total number of peptide bonds available for proteolytic hydrolysis (8.6 meq/g), and *α* is the average degree of dissociation of the amino groups in the protein substrate, and it was determined with:

(3) $$\alpha =\frac{10^{pH-pK}}{1+10^{pH-pK}}.$$

The *pK* value is dependent on the temperature of hydrolysis (in K degrees) and can be estimated according to the expression:

(4) $$pK=7.8+2400\left(\frac{298-T}{298T}\right).$$

Finally, the kinetics data of the *S. canicula* muscle hydrolysis (*H*) were modelled by means of the Weibull equation [24]:

(5) $$H=H_{m}\left\{1-\exp\left[-\ln2{\left(\frac{t}{\tau }\right)}^{\beta }\right]\right\}\;\mathrm{with}\;v_{m}=\frac{H_{m}\beta \ln2}{2\tau },$$

where *H* is the degree of hydrolysis (%); *t* is the time of hydrolysis (min); *H_m_* is the maximum degree of hydrolysis (%); *β* is a parameter related to the maximum slope of muscle hydrolysis (dimensionless); *τ* is the time required to achieve the semi-maximum degree of hydrolysis (min) and *v_m_* is the maximum hydrolysis rate at the *τ*-time (% min^−1^).

### 3.4. Antihypertensive Activities and Angiotensin I-Converting Enzyme (ACE) Inhibition Assay

The antihypertensive activity of *S. canicula* hydrolysates was determined using *N*-[3-(2-Furyl) acryloyl]-l-phenylalanyl-glycyl-glycine (FAPGG) as substrate according the modifications reported by Estévez et al. [28] ACE-inhibitory activity (*IACE*) of hydrolysates was calculated as a function of the average slope of decrease in Absorbance with time and expressed as percentage inhibition of the enzyme, according to the following expression:

(6) $$IACE\left(\%\right)=\left(1-\frac{rA_{h}}{rA_{c}}\right)\times 100,$$

where *IACE* is the ACE-inhibitory capacity (%), *rA_h_* is the slope of decrease in Absorbance at 340 nm in the presence of inhibitor (hydrolysate), and *rAc* is the slope decrease in Absorbance at 340 nm in the absence of inhibitor (control). The protein-hydrolysate concentration that generates a 50% of *IACE* (*IC*_50_) was calculated by fitting the dose-response relationship between *IACE* vs. hydrolysate to a Weibull equation [37]:

(7) $$IACE=K\left\{1-\exp\left[-\ln2{\left(\frac{C}{IC_{50}}\right)}^{a}\right]\right\}$$

where *K* is the maximum *IACE* (%), *C* is the protein-hydrolysate concentration (μg/mL), *IC*_50_ is the concentration for semi-maximum *IACE* (μg/mL), and *a* is the form parameter related to the maximum slope of the function (dimensionless)*.*

### 3.5. Antioxidant Activity Determinations

#### 3.5.1. 1,1-Diphenyl-2-Picryhydrazyl (DPPH) Radical-Scavenging Capacity

The antioxidant activity as radical-scavenging capacity was determined with DPPH as a free radical by the microplate method described by Prieto et al. [38]. The DPPH activity was calculated as a percentage of DPPH discoloration using the equation:

(8) $$DPPH\left(\%\right)=\frac{{\left(A_{control}\right)}_{t=1h}-{\left(A_{hydrolysate}\right)}_{t=1h}}{{\left(A_{control}\right)}_{t=1h}}\times 100,$$

where *A_sample_* is the Absorbance at 515 nm of the DPPH in the presence of the hydrolysate, and *A_control_* is the Absorbance at 515 nm of the DPPH solution in its absence.

#### 3.5.2. ABTS Bleaching Method

The ABTS (2,2′-azinobis-(3-ethyl-benzothiazoline-6-sulphonic acid) radical scavenging activities were evaluated according the protocol previously reported [38]. The ABTS activity was calculated as a percentage of ABTS discoloration using the equation:

(9) $$ABTS\left(\%\right)=\frac{{\left(A_{control}\right)}_{t=2h}-{\left(A_{hydrolysate}\right)}_{t=2h}}{{\left(A_{control}\right)}_{t=2h}}\times 100,$$

where *A_sample_* is the absorbance at 414 nm of the ABTS in the presence of the hydrolysate, and *A_control_* is the absorbance at 414 nm of the ABTS solution in its absence.

#### 3.5.3. *β*-Carotene Bleaching Method

The kinetics of the *β*-carotene (*β*-C) bleaching assay were performed according the protocol described for its use in microplate spectrophotometer developed by Prieto et al. [39].

#### 3.5.4. Crocin Bleaching Method

The kinetics of the crocin (Cr) bleaching assay were based on the protocol optimized by Prieto et al. [40] using crocin and 2,2′-azobis-2-amidinopropane (AAPH) as reagents and carried out in microplate spectrophotometer.

In these last two methods the kinetics of reaction were performed in triplicate. For each series, reversed curves were obtained by subtracting the Absorbance at time *t* from the Absorbance value at time 0. The area under the curves (AUC) can be calculated by the following function [37]:

(10) $$AUC=\left(y_{0}+2y_{1}+2y_{2}+\ldots +2y_{n-2}+2y_{n-1}+y_{n}\right)\frac{\Delta t}{2},$$

where *y*_0_ to *y_n_* are the *n* + 1 *y*-values defining the curve, and Δ*t* is the sampling interval (min).

Calculated areas of controls concentrations (Trolox for Cr and BHT for *β*-C) were fitted by linear regression. Calculated areas of hydrolysate dilutions were plotted against controls (equivalents) and the antioxidant activities (as equivalents in μg of BHT or Trolox per mL of hydrolysate) were defined by means of the *EC*_50_ values obtained by fitting the data of equivalents versus sample concentrations to a similar Weibull equation [7] (but replacing *IC*_50_ by *EC*_50_).

### 3.6. Numerical and Statistical Analyses

Fitting procedures and parametric estimations calculated from the non-linear equations were carried out by minimising the sum of quadratic differences between the observed and model-predicted values and using the non-linear least-squares (quasi-Newton) method provided by the macro-‘Solver’ of the Microsoft Excel spreadsheet. Confidence intervals from the parametric estimates (Student *t*-test) and consistence of mathematical models (Fisher *F*-test) were evaluated by “SolverAid” macro (Levie’s Excellaneous website: http://www.bowdoin.edu/~rdelevie/excellaneous).

## 4. Conclusions

In the present report, we have optimised the production of FPH with potentially bioactive peptides, including antioxidant and antihypertensive properties, from the *S. canicula* muscle by enzymatic hydrolysis using the response surface methodology. The optimal conditions for the highest proteolysis were established in 60.8 °C/pH8.9 and 64.6 °C/pH9.4 for Esperase and Alcalase, respectively. No hydrolysis was, however, detected when using Protamex. The lower *IC*_50_ values (higher ACE activity) were achieved with hydrolysates obtained at 59.4 °C for Alcalase and 44.6 °C for Esperase, and any level of *pH*. In such conditions, the Esperase hydrolysate was more active than the Alcalase one. For antioxidants, ABTS and DPPH activities were maximized in the range of 55–63.1 °C and *pH* of 8.0–9.0. Nevertheless, further studies should be conducted in order to characterise and isolate the bioactive peptides that produce the best antioxidant and antihypertensive activities.

## Figures and Tables

**Table 1:** Parametric estimations corresponding to hydrolysis data modelling by Weibull equation of the experimental conditions studied (Table S1). Independent variables are expressed in natural values in brackets. Numerical values of the parameters are shown with their confident intervals. Determination coefficients (R^2^) and *p*-values from Fisher *F*-test are also summarized. NHD: non hydrolysis detected.

	Experimental Conditions	*H_m_* (%)	*v_m_* (% min^−1^)	τ (min)	*β*	R^2^	*p*-Value
Alcalase	*T*:−1 (37.3 °C)/*pH*:−1 (6.9)	11.39 ± 0.32	0.068 ± 0.006	42.41 ± 2.95	0.73 ± 0.05	0.982	<0.001
	*T*:1 (72.7 °C)/*pH*:−1 (6.9)	13.52 ± 0.01	0.811 ± 0.012	4.08 ± 0.09	0.71 ± 0.01	0.999	<0.001
	*T*:−1 (37.3 °C)/*pH*:1 (11.1)	NHD	NHD	NHD	NHD	NHD	NHD
	*T*:1 (72.7 °C)/*pH*:1 (11.1)	29.26 ± 1.34	0.063 ± 0.005	122.2 ± 11.6	0.76 ± 0.03	0.998	<0.001
	*T*:−1.41 (30.0 °C)/*pH*:0 (9.0)	12.57 ± 0.29	0.053 ± 0.003	75.53 ± 3.22	0.92 ± 0.04	0.991	<0.001
	*T*:1.41 (80.0 °C)/*pH*:0 (9.0)	12.79 ± 9.30	0.002 ± 0.001	826.5 (NS)	0.34 ± 0.06	0.966	<0.001
	*T*:0 (55.0 °C)/*pH*:−1.41 (6.0)	5.61 ± 0.01	1.67 ± 0.30	0.44 ± 0.11	0.38 ± 0.03	0.974	<0.001
	*T*:0 (55.0 °C)/*pH*:1.41 (12.0)	NHD	NHD	NHD	NHD	NHD	NHD
	*T*:0 (55.0 °C)/*pH*:0 (9.0)	21.66 ± 0.39	0.125 ± 0.007	31.56 ± 1.59	0.53 ± 0.02	0.994	<0.001
	*T*:0 (55.0 °C)/*pH*:0 (9.0)	24.25 ± 0.27	0.207 ± 0.009	17.06 ± 0.62	0.42 ± 0.01	0.997	<0.001
	*T*:0 (55.0 °C)/*pH*:0 (9.0)	29.19 ± 5.17	0.042 ± 0.025	95.71 ± 64.35	0.39 ± 0.05	0.971	<0.001
	*T*:0 (55.0 °C)/*pH*:0 (9.0)	29.22 ± 4.42	0.056 ± 0.038	55.45 ± 40.32	0.31 ± 0.04	0.975	<0.001
	*T*:0 (55.0 °C)/*pH*:0 (9.0)	21.85 ± 2.50	0.089 ± 0.042	30.35 ± 14.03	0.36 ± 0.05	0.951	<0.001
Esperase	*T*:−1 (37.3 °C)/*pH*:−1 (6.6)	NHD	NHD	NHD	NHD	NHD	NHD
	*T*:1 (72.7 °C)/*pH*:−1 (6.6)	NHD	NHD	NHD	NHD	NHD	NHD
	*T*:−1 (37.3 °C)/*pH*:1 (9.4)	12.95 ± 0.13	0.149 ± 0.007	24.13 ± 1.26	0.80 ± 0.05	0.972	<0.001
	*T*:1 (72.7 °C)/*pH*:1 (9.4)	30.0 ± 18.04	0.004 (NS)	604.8 (NS)	0.25 ± 0.05	0.969	<0.001
	*T*:−1.41 (30.0 °C)/*pH*:0 (8.0)	5.41 ± 0.08	0.041 ± 0.003	122.5 ± 2.62	2.66 ± 0.19	0.978	<0.001
	*T*:1.41 (80.0 °C)/*pH*:0 (8.0)	11.73 ± 0.02	1.43 ± 0.06	2.10 ± 0.16	0.74 ± 0.05	0.981	<0.001
	*T*:0 (55.0 °C)/*pH*:−1.41 (6.0)	NHD	NHD	NHD	NHD	NHD	NHD
	*T*:0 (55.0 °C)/*pH*:1.41 (10.0)	20.54 ± 0.05	0.532 ± 0.012	8.43 ± 0.29	0.63 ± 0.02	0.993	<0.001
	*T*:0 (55.0 °C)/*pH*:0 (8.0)	24.45 ± 0.18	0.249 ± 0.007	16.20 ± 0.40	0.48 ± 0.01	0.997	<0.001
	*T*:0 (55.0 °C)/*pH*:0 (8.0)	29.42 ± 0.54	0.209 ± 0.014	22.04 ± 0.93	0.45 ± 0.02	0.993	<0.001
	*T*:0 (55.0 °C)/*pH*:0 (8.0)	25.25 ± 0.23	0.281 ± 0.010	14.79 ± 0.47	0.48 ± 0.01	0.995	<0.001
	*T*:0 (55.0 °C)/*pH*:0 (8.0)	29.78 ± 0.29	0.318 ± 0.012	15.67 ± 0.52	0.48 ± 0.01	0.995	<0.001
	*T*:0 (55.0 °C)/*pH*:0 (8.0)	27.83 ± 0.21	0.322 ± 0.010	14.52 ± 0.40	0.49 ± 0.01	0.996	<0.001

**Table 2:** Empirical models describing the combined effect of temperature (*T*) and *pH* on the enzyme hydrolysis parameters and the antihypertensive activities (ACE-inhibitory activity (*IACE*) in % and *IC*_50_ in μg/mL) of *S. canicula* muscle hydrolysates. Optima values of the two variables (*T_opt_* and *pH_opt_*) to obtain the maximum responses (*Y_max_*) from the empirical equations are summarized. The coefficients of determination R^2^ and the results of Fisher *F*-tests (*F1*, *F2*, *F3*, and *F4*) are also shown. S: significant; NS: non-significant.

Enzyme/Activity	Polynomial Equations	R^2^	Fisher *F*-Test	*T_opt_* (°C)	*pH_opt_*	*Y_max_*
Alcalase/Hydrolysis	*H_m_* (%) = 25.22 + 3.97 *T* + 6.78 *T pH* − 4.82 *T*^2^ − 9.79 *pH*^2^	0.698	*F1*: S; *F2*: S; *F3*: NS; *F4*: NS	64.6	9.40	26.3%
	*v_m_* (% min^−1^) = 0.104 + 0.092 *T* − 0.398 *pH* − 0.170 *T pH* − 0.088 *T*^2^ + 0.318 *pH*^2^	0.693	*F1*: S; *F2*: S; *F3*: NS; *F4*: NS	53.9	10.29	1.6% min^−1^
Esperase/Hydrolysis	*H_m_* (%) = 27.34 + 3.25 *T* + 9.02 *pH* + 4.26 *T pH* − 9.09 *T*^2^ − 8.23 *pH*^2^	0.943	*F1*: S; *F2*: S; *F3*: S; *F4*: S	60.8	8.90	30.7%
	*v_m_* (% min^−1^) = 0.277 + 0.228 *T* + 0.113 *pH* + 0.115 *T*^2^ − 0.122 *pH*^2^	0.413	*F1*: S; *F2*: S; *F3*: NS; *F4*: NS	80	8.65	0.90% min^−1^
Alcalase/Antihypertensive	*IACE* (%) = 74.51 + 7.61 *T* + 3.48 *pH* − 6.03 *T*^2^ − 6.50 *pH*^2^	0.746	*F1*: S; *F2*: S; *F3*: NS; *F4*: NS	66.2	11.5	90.7%
	* IC_50_ (μg/mL) = 117.42 − 23.61 *T* + 47.81 *T*^2^	0.739	*F1*: S; *F2*: S; *F3*: NS; *F4*: NS	59.4	non effect	114.5 μg/mL
Esperase/Antihypertensive	*IACE* (%) = 74.42 − 3.87 *T*	0.496	*F1*: S; *F2*: S; *F3*: NS; *F4*: NS	30.0	non effect	79.9%
	* IC_50_ (μg/mL) = 111.41 + 94.34 *T* + 80.12 *T*^2^	0.583	*F1*: S; *F2*: S; *F3*: NS; *F4*: NS	44.6	non effect	83.64 μg/mL

**Table 3:** Empirical models describing the combined effect of temperature (*T*) and *pH* on the antioxidant activities (*β*-carotene: *β*-C, crocin: Cr, DPPH and ABTS methods) of *S. canicula* muscle hydrolysates. Optima values of the two variables (*T_opt_* and *pH_opt_*) to obtain the maximum responses (*Y_max_*) from the empirical equations are summarized. The coefficients of determination R^2^ and the results of Fisher *F*-test (*F1, F2, F3 and F4*) are also shown. S: significant; NS: non-significant; Tr: Trolox.

Enzyme	Polynomial Equations	R^2^	Fisher *F*-Test	*T_opt_* (°C)	*pH_opt_*	*Y_max_*
Alcalase	*β-C* (μg BHT/mL) = 0.08 + 0.28 *pH* − 0.09 *T*^2^ + 0.26 *pH*^2^	0.680	*F1*: S; *F2*: S; *F3*: NS; *F4*: NS	55.0	12.0	1.09 μg BHT/mL
	*Cr* (μg Trolox/mL) *=* 1.63 − 0.95 *T* + *0.98 pH* − 1.53 *T pH*	0.543	*F1*: S; *F2*: S; *F3*: NS; *F4*: NS	30.0	12.0	7.96 μg Tr/mL
	*DPPH* (%) *=* 12.07 + 1.44 *T* − 1.57 *T*^2^ − 2.68 *pH*^2^	0.833	*F1*: S; *F2*: S; *F3*: NS; *F4*: NS	63.1	9.0	12.4%
	*ABTS* (%) *=* 5.10 + 1.24 *T pH* − 0.85 *T*^2^ − 0.82 *pH*^2^	0.578	*F1*: S; *F2*: S; *F3*: NS; *F4*: NS	55.0	9.0	5.1%
Esperase	*Cr* (μg Trolox/mL) *=* 2.23 − 0.62 *T*^2^ − 0.61 *pH*^2^	0.625	*F1*: S; *F2*: S; *F3*: NS; *F4*: NS	55.0	8.0	2.23 μg Tr/mL
	*DPPH* (%) *=* 16.02 − 2.31 *T pH* − 2.26 *T*^2^ − 2.49 *pH*^2^	0.840	*F1*: S; *F2*: S; *F3*: NS; *F4*: NS	55.0	8.0	16.0%
	*ABTS* (%) *=* 7.30 − 1.44 *T*^2^ − 1.64 *pH*^2^	0.752	*F1*: S; *F2*: S; *F3*: NS; *F4*: NS	55.0	8.0	7.3%

## Supplementary Materials

The following are available online at www.mdpi.com/1660-3397/15/10/306/s1, Figure S1: Proteolysis kinetics of *S. canicula* muscle wastes mediated by Esperase, Figure S2: Proteolysis kinetics of *S. canicula* muscle wastes mediated by Alcalase, Table S1: Experimental domain values, Table S2: Experimental results for hydrolysates obtained by Alcalase, Table S3: Experimental results for hydrolysates obtained by Esperase.
