# Supplementary material for: Production of Fish Protein Hydrolysates from Scyliorhinus canicula Discards with Antihypertensive and Antioxidant Activities by Enzymatic Hydrolysis and Mathematical Optimization Using Response Surface Methodology
Source: Mar Drugs. 2017 Oct 10;15(10):306. doi: 10.3390/md15100306 (PMC5666414; doi:10.3390/md15100306)
Supplement: Supplementary file 1 [file marinedrugs-15-00306-s001.pdf]

Figures_Supplementary Material

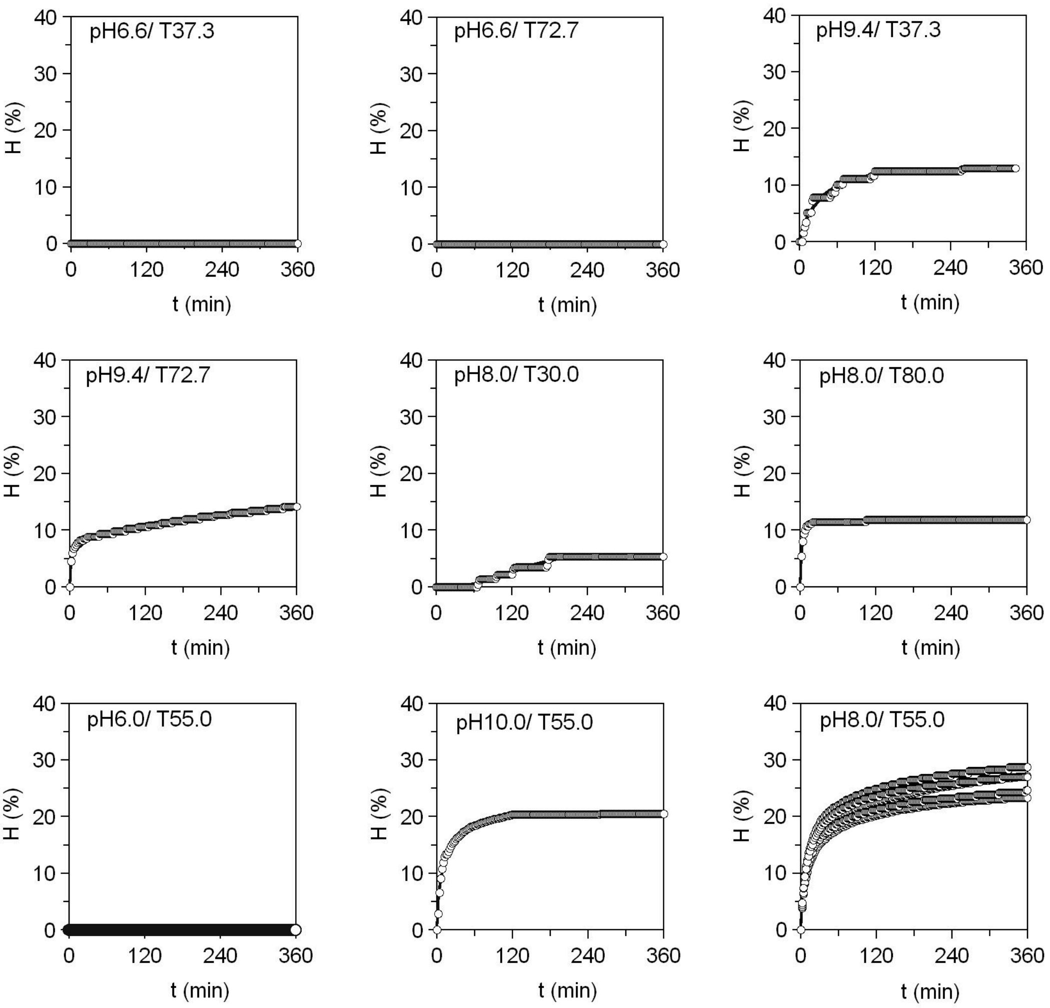

Figure S1. Proteolysis kinetics of *S. canicula* muscle wastes mediated by Esperase under the experimental conditions specified in the factorial design summarized in Table S1 (Supplementary Material) (*T* in ºC). Experimental data (symbols) were fitted to Weibull equation [1] (lines).

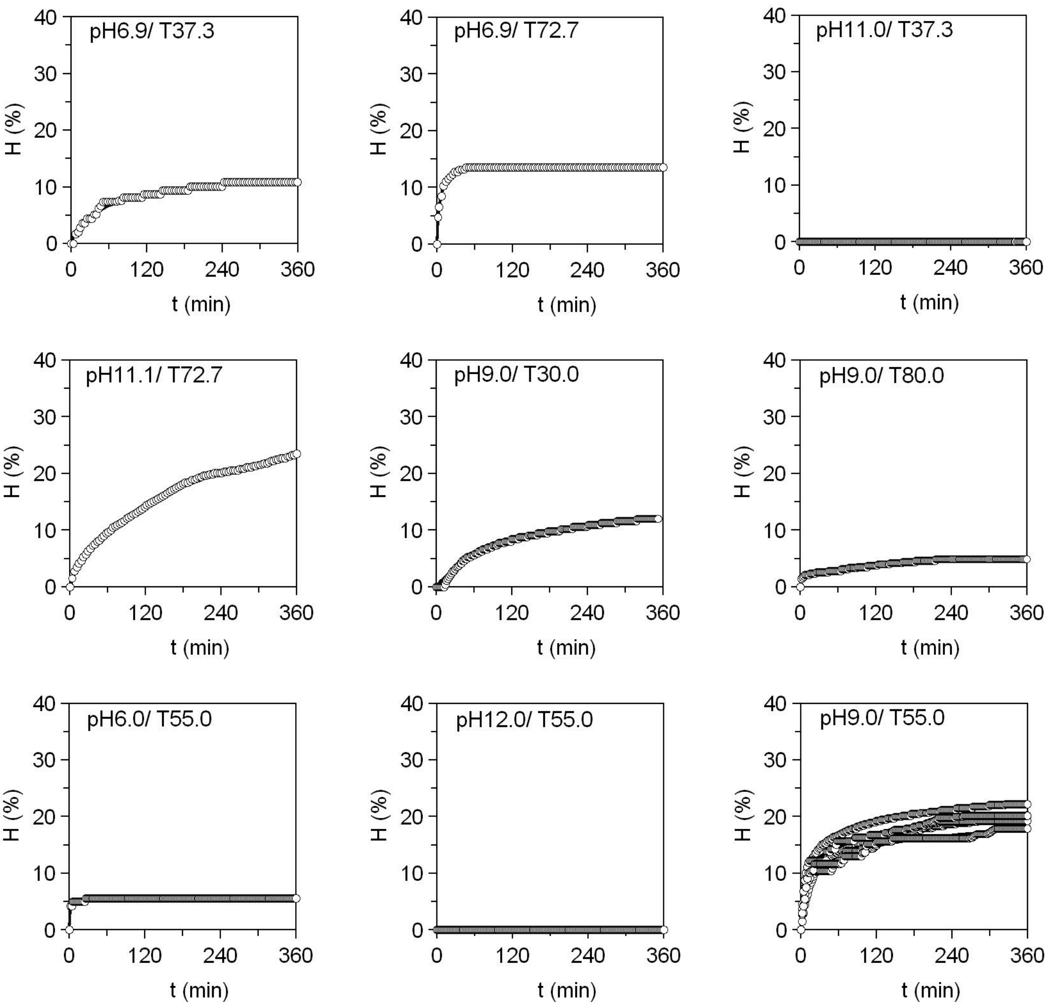

Figure S2. Proteolysis kinetics of *S. canicula* muscle wastes mediated by alcalase under the experimental conditions defined by the factorial design summarized in Table S1 (*T* in ºC). Experimental data (symbols) were fitted to Weibull equation [1] (lines).

TABLES supplementary material

Table S1. Experimental domain values and codification assignment of independent variables used for factorial rotatable design regarding commercial proteases catalysis.

	Natural values					
	Alcalase		Esperase		Protamex	
**Coded values**	**pH**	**T (ºC)**	**pH**	**T (ºC)**	**pH**	**T (ºC)**
-1.41	6.0	30.0	6.0	30.0	4.0	30.0
-1	6.9	37.3	6.6	37.3	4.9	37.3
0	9.0	55.0	8.0	55.0	7.0	55.0
+1	11.1	72.7	9.4	72.7	9.1	72.7
+1.41	12.0	80.0	10.0	80.0	10.0	80.0

Codification: $V_c = (V_n - V_0)/\Delta V_n$ Decodification: $V_n = V_0 + (\Delta V_n \times V_c)$
V_0 = natural value in the centre of the domain
V_n =natural value of the variable to codify
ΔV_n = increment of V_n for unit of V_c

Table S2. Summary of the experimental (IACE, IC_{50}, DPPH, ABTS, β-C and Cr) and expected values ($IACE_p$, IC_{50p}, $DPPH_p$, $ABTS_p$, β-C_p and Cr_p) for the antihypertensive and antioxidant activities produced in the *S. canicula* hydrolysates obtained by alcalase. X_1: Temperature (ºC) and X_2: pH. Natural values of experimental conditions are in brackets.

Independent variables		Antihypertensive activities				Antioxidant activities							
X_1: *T*	X_2: *pH*	IACE (%)	$IACE_e$ (%)	IC_{50} (µg/mL)	IC_{50e} (µg/mL)	DPPH (%)	$DPPH_e$ (%)	ABTS (%)	$ABTS_e$ (%)	β-C (µg/mL)	β-C_e (µg/mL)	Cr (µg/mL)	Cr_e (µg/mL)
-1 (37.3)	-1 (6.9)	73.9	63.9	119.6	188.8	7.50	6.39	5.90	4.67	0.056	-0.025	0.97	0.07
1 (72.7)	-1 (6.9)	86.0	79.1	119.2	141.6	9.55	9.27	1.32	2.20	0.019	-0.025	0.33	1.22
-1 (37.3)	1 (11.1)	73.4	70.9	190.7	188.8	7.39	6.39	3.62	2.20	0.197	0.532	7.42	5.09
1 (72.7)	1 (11.1)	79.9	86.1	143.7	141.6	9.26	9.27	3.98	4.67	0.136	0.532	0.67	0.13
-1.41 (30)	0 (9)	44.2	51.8	279.6	245.8	5.68	6.93	1.69	3.41	0.052	-0.096	0.50	2.97
1.41 (80)	0 (9)	74.1	73.3	179.5	179.2	11.03	10.98	4.58	3.41	0.061	-0.096	0.34	0.28
0 (55)	-1.41 (6)	71.9	82.5	136.0	117.4	6.69	6.75	3.52	3.47	0.057	0.208	0.42	0.24
0 (55)	1.41 (12)	96.3	92.3	113.3	117.4	5.61	6.75	2.88	3.47	1.448	0.993	1.15	3.01
0 (55)	0 (9)	71.3	74.5	109.3	117.4	11.51	12.07	5.42	5.10	0.151	0.079	1.81	1.63
0 (55)	0 (9)	77.1	74.5	106.3	117.4	12.74	12.07	4.77	5.10	0.075	0.079	0.79	1.63
0 (55)	0 (9)	73.0	74.5	133.5	117.4	10.27	12.07	5.39	5.10	0.071	0.079	0.64	1.63
0 (55)	0 (9)	76.4	74.5	115.5	117.4	13.30	12.07	4.30	5.10	0.075	0.079	2.91	1.63
0 (55)	0 (9)	74.9	74.5	122.2	117.4	12.57	12.07	5.61	5.10	0.017	0.079	2.00	1.63

Table S3. Summary of the experimental (IACE, IC_{50}, DPPH, ABTS, β-C and Cr) and expected values ($IACE_p$, IC_{50p}, $DPPH_p$, $ABTS_p$, β-C_p and Cr_p) for the antihypertensive and antioxidant activities produced in the *S. canicula* hydrolysates obtained by esperase. X_1: Temperature (ºC) and X_2: pH. Natural values of experimental conditions are in brackets.

Independent variables		Antihypertensive activities				Antioxidant activities							
X_1: *T*	X_2: *pH*	IACE (%)	$IACE_e$ (%)	IC_{50} (µg/mL)	IC_{50e} (µg/mL)	DPPH (%)	$DPPH_e$ (%)	ABTS (%)	$ABTS_e$ (%)	β-C (µg/mL)	β-C_e (µg/mL)	Cr (µg/mL)	Cr_e (µg/mL)
-1 (37.3)	-1 (6.9)	75.2	77.9	98.9	97.2	10.04	8.97	5.81	4.22	0.035	0.034	2.02	1.00
1 (72.7)	-1 (6.9)	70.8	70.9	153.5	285.9	13.96	13.58	2.91	4.22	0.050	0.034	0.83	1.00
-1 (37.3)	1 (11.1)	68.9	77.9	181.5	97.2	13.25	13.58	4.50	4.22	0.038	0.034	0.53	1.00
1 (72.7)	1 (11.1)	73.5	70.9	153.3	285.9	7.94	8.97	4.24	4.22	0.042	0.034	0.45	1.00
-1.41 (30)	0 (9)	83.5	79.3	87.5	137.7	13.00	11.53	3.17	4.43	0.045	0.034	1.00	1.00
1.41 (80)	0 (9)	63.6	69.5	602.5	403.7	10.02	11.53	5.38	4.43	0.034	0.034	1.10	1.00
0 (55)	-1.41 (6)	70.6	74.4	237.7	111.4	10.20	11.07	4.21	4.04	0.057	0.034	1.07	1.02
0 (55)	1.41 (12)	71.2	74.4	249.4	111.4	11.89	11.07	3.57	4.04	0.003	0.034	1.07	1.02
0 (55)	0 (9)	73.8	74.4	180.0	111.4	14.50	16.02	7.42	7.30	0.037	0.034	2.17	2.23
0 (55)	0 (9)	74.2	74.4	134.3	111.4	16.26	16.02	6.77	7.30	0.036	0.034	3.00	2.23
0 (55)	0 (9)	73.9	74.4	90.2	111.4	17.26	16.02	*8.39*	7.30	0.013	0.034	2.45	2.23
0 (55)	0 (9)	77.5	74.4	101.3	111.4	15.85	16.02	6.30	7.30	0.055	0.034	1.55	2.23
0 (55)	0 (9)	72.7	74.4	49.6	111.4	16.22	16.02	7.61	7.30	0.029	0.034	2.00	2.23
